# Outlines of Zoonosological Tables

**Published:** 1817-12

**Authors:** Thomas Parkinson


### *For the London Medical and Physical Journal.*

### *Outlines of Zoonosological Tables;* by Dr. Thomas Parkinson.

THE plan I have adopted for the improvement of medical science consists of an entirely new Nomenclature in every department; and each department is provided with its own peculiar Nomenclature.

Each department of medical science is limited to its natural province.

1. Anatomy is limited to description and demonstration of the distinguishable parts and peculiarities of the animal frame.

2. Physiology is limited to description and demonstration of official capacities and modes of function in health.

3. Nosology is limited to description and demonstration of those states of the animal and vital actions which are between health and death, and those arrangements of organization, or function, which are the resulting consequences of primary morbid affections.

4. Therapeutics is limited to the description and demonstration of indications, and agents employed for the prevention, mitigation, and removal of diseases.

The technical terms of anatomy are such as express the peculiar constructions and compositions of the distinguishable parts of the body.

CLASSES—EXAMPLES—SOLIDS.

1. Myeleuthytōna. From Μυελος, the medullary substance, and ευθυς, straight; signifying straight fibres with brain. The characteristics of nerve.

2. Hæmeuthytōna. From Αιμα, blood, and ευθυς, straight; signifying straight fibres with blood. The characteristics of muscle.

3. Helasmata. From Ελασμα, laminated fibre. The characteristic of elastic fibre.

4. Enyphanta. From Ενυφαινω, to intertex, intertexed fibre. The characteristic of membrane.

These are all the classes of solids, each of which may be considered an abstract solid. But each class has its genera accordingly as it is associated with another class, or with other substances,* or is thrown into varied forms or shapes.

Hence Myeleuthytona has two genera:—

1. Myeleuthytona Hæmeuthytodes. An association of nerve with muscle. As perceiving organs and the brain within the head.
2. Myeleuthytona Enyphantodes. An association of nerve with its investing membrane. As the nervous cord.

Hæmeuthytona has two genera:—

1. Hæmeuthytona Stereoses. Solid muscle. Muscles in general.
2. Hæmeuthytona Antrodes. Chambered muscle. Muscles of the heart.

Helasmata has three genera.:—

1. Helasmata Stereoses. Solid elastic fibre. Elastic ligaments.
2. Helasmata Siphonodes. Tubulated elastic fibre. Artery.
3. Helasmata Siphogyrodes. Tubulated and convoluted elastic fibre. Gland.

Enyphanta has ten genera:—

1. Enyphanta Platynodes. Simple broad membrane. Dura and pia maters, pleura, peritonæum, fascia, &c.

---

* N.B, In genera 5, 6, 7, 8, 9, Enyphanta secretas, its own associates.

2. Enyphanta

2. Enyphanta Siphonodes. Tubulated membrane. Vein, duct, &c.
3. Enyphanta Helasmatodes. An association of membrane with elastic fibre. The substance of the lungs, corpora, penis, &c.
4. Enyphanta Hæmeuthytodes. An association of membrane with muscle. The intestinal canal, &c.
5. Enyphanta Collodes. An association of membrane with albumen. Cartilage.
6. Enyphanta Siphocollodes. The same association tubulated. The annular cartilages of the trachea and bronchia, the cartilaginous meatus of the ear, &c.
7. Enyphanta Coniodes. An association of membrane with lime. Bone.
8. Enyphanta Siphoconiodes. The same association tubulated. Large bones of the extremities, the bony meatus of the ear.
9. Enyphanta Steatodes. An association of membrane with fat. Adipose membrane.
10. Enyphanta Siphogyrodes. Membrane tubulated and convoluted, to form a gland. The liver.

FLUIDS.

Fluids have two classes:—

1. Perirrotica. From Πεϱιϱϱεω, to circulate. Fluids circulating in vessels.

2. Limnetica. From Λιμνη, stagnant. Fluids not circulating in vessels.

The first class has four genera, but no varieties.

1. Hæma. From Αιμα, blood. Perfect arterious blood.

2. Hæmatodes. From Αιματωδης, bloody. Imperfect blood; venous blood.

3. Diaphanodes. From Διαφανης, limpid, or diaphanous. Lymph.

4. Galatodes. From Γαλατωδης, milky. Chyle.

The second class has three genera, with their respective varieties:—

1. Phylacteria. From φυλασσω, to imprison. Incysted fluid.

2. Diakosia. From Διαχεω, to disperse. Interstitial fluid.

3. Exothisma. From Εξοθεω, to expel. Expelled fluid.

The first genus has five varieties:—

1. Urodes. From ϐϱον. Urine.

2. Cholodes. From χολη. Bile.

3. Spermatodes. From Σπεϱμα. Semen.

4. Ophthalmodes. From Οφθαλμος. The eye.

5. Copriodes. From Κοπϱος. Dung.

The second genus has two varieties:—

1. Hydalodes. From Υδωϱ. Water.

2. Blennodes. From βλεννα. Mucilage.

The third genus has two varieties:—

1. Hydalodes. From Υδωϱ. Water.

2. Blennodes. From βλεννα. Mucilage.

The three large cavities are denominated Teleoses, from Τελεωσις, an elaboratory or workshop.

There are three Teleoses:—

1. Phreneteleosis. From φϱην, the mind, and τελεωσις, the elaboratory of the mind. The encephalon.

2. Hæmateleosis. From Αιμα, blood, and τελεωσις, the elaboratory of the blood. The thorax.

3. Tropheteleosis. From τϱοφη, nourishment, and τελεωσις, the elaboratory of nourishment. The abdomen.

 This

This classification and arrangement affords a convenient opportunity of affixing the proper physiology to each of the genera of solids, and to each of the genera and varieties of fluids.

Myeleuthytona Hæmeuthytodes. Æsthesis. Sense.
Myeleuthytona Enyphantodes. Æsthephoresis. Carriers of sense.
Hæmeuthytona Stereoses. Kinesis. Direct or positive action.
Hæmeuthytona Antrodes. Kinesis. Direct or positive action.
Helasmata Stereoses. Apokinesis. Re-action.
Helasmata Siphonodes. Siphapokinesis. Tubular re-action.
Helasmata Siphogyrodes. Eccrisis. Secretion.
Enyphanta Platynodes. Parenthesis. Interposition.
Enyphanta Siphonodes. Siphoparenthesis. Tubular interposition.
Enyphanta Helasmatodes. Parenthapokinesis. Interposition and re-action.
Enyphanta Hæmeuthytodes. Parenthisystolesis. Interposition and constriction.
Enyphanta Collodes. Diastolesis. Dilatation—preserving distances.
Enyphanta Siphocollodes. Siphodiastolesis. Keeping tubes perpetually open.
Enyphanta Coniodes. Acamptosis. Inflexibility.
Enyphanta Siphoconiodes. Acamptosis. Inflexibility.
Enyphanta Steatodes. Morphosis. Giving form or shape.
Enyphanta Siphogyrodes. Eccrisis. Secretion.

FLUIDS.

Hæma. Stereotrophisis. Nourishing solids.
Hæmatodes. Hæmathemeleosis. The foundation or Basis of Hæma.
Diaphanodes. Hæmaleptosis. The vehicle and diluter of blood.
Galatodes. Hæmatrophisis. Nourishing the blood.
Urodes. Akrestophoresis. Carrying away useless materials.
Chalodes. Pepsis. Digestion.
Spermatodes. Paraphusis. Procreation.
Ophthalmodes. Diastolesis. Preserving proper distances.
Copriodes. Akrestophoresis. Carrying away useless materials.
Hydalodes. Hygrosis. Moistening and washing.
Blennodes. Blennosis. Lubrication.

TELEOSES.

Phreneteleosis. Phrenepoesis. Fabrication of the mind.
Hæmateleosis. Hæmapoesis. Fabrication of the blood.
Tropheteleosis. Trophepoesis. Fabrication of nourishment.

It is pretty clear, from the above Prospectus Anatomico-Physiologicus, that the functions of the various parts of the body arise, as inevitable consequences of organization and composition, of which two of those functions are the essential attributes of animal life—Sensation and Motion, (Æsthesis and Kinesis. And, in considering the nature of the human mind, it is essentially necessary to keep these two faculties distinct, otherwise we shall get bewildered: indeed, it appears to me evident, that, without an improved and appropriate Nomenclature, we shall remain without a solid system of medical science; and so long as nerve, muscle, tendon, artery, &c. are suffered to remain as anatomical denominators, whilst physiology is unprovided with such technical cal terms as denote identical faculties; whilst diseases are denominated by their symptoms instead of their identities; and, whilst medical phraseology is so loose and whimsical, it cannot be expected that any splendid attainments will be acknowledged, or that the veil will be pierced which conceals the composition and fabric of the human mind.

The analysis of the human mind will be the first subject of my next paper.

*Leicester; October* 14, 1817.

