# Errata

**Published:** 1818-01

**Authors:** 


ERRATA.

In our last, page 458, in two places, *for* decemus *read* dicemus.

